# 基于机器学习的全氟及多氟化合物电离效率预测模型开发及其在半定量分析中的应用

**DOI:** 10.3724/SP.J.1123.2025.02012

**Published:** 2026-04-08

**Authors:** Shenzheng SUN, Yaoyao LI, Yan GAO, Kangcong LI, Zhi CHEN, Xiuqin LI, Qinghe ZHANG

**Affiliations:** 1.中国计量大学材料与化学学院，浙江 杭州 310018; 1. China Jiliang University，College of Materials and Chemistry，Hangzhou 310018，China; 2.中国计量科学研究院化学计量 与分析科学研究所，北京 100029; 2. Division of Chemical Metrology and Analytical Science，National Institute of Metrology，China，Beijing 100029，China

**Keywords:** 全氟及多氟烷基化合物, 定量构效关系, 机器学习, 半定量预测模型, per- and polyfluoroalkyl substances （PFASs）, quantitative structure-activity relationship （QSAR）, machine learning, semi-quantitative predictive model

## Abstract

全氟和多氟烷基化合物（PFASs）具有持久性、生物累积性和潜在毒性，在环境科学和食品安全等领域备受关注。尽管基于高分辨率质谱（HRMS）的PFASs筛查方法发展迅速，但PFASs种类繁多，无标准品的PFASs定量困难。本研究对50种PFASs进行液相色谱-高分辨质谱分析，计算其电离效率（IE），并基于机器学习建立PaDEL分子描述符与PFASs电离效率的定量构效关系（QSAR）预测模型，该模型的构建能够预测无标准品PFASs的电离效率，通过预测的电离效率进行PFASs的半定量预测。研究采用递归特征消除（RFE），从1 444个PaDEL描述符中选取了18个特征变量；比较了弹性网络线性回归、随机森林和极致梯度提升（XGBoost）3种不同算法所建立的QSAR模型性能。结果表明，XGBoost模型性能最优，其训练集的均方根误差（RMSE）为0.052 1，决定系数（*R* ^2^）为0.992 0；测试集的RMSE为0.118 4，*R* ^2^为0.871 3。50种PFASs预测的IE误差在1.67倍以内，中位值为1.04倍，RMSE为1.06。采用预测的电离效率值对不同梯度浓度的PFASs标准溶液进行半定量预测来验证模型性能，浓度预测误差倍数为0.12~4.90倍，中位值为0.96倍，RMSE为0.94；且随着溶液浓度升高，浓度半定量预测的准确度提高。将该电离效率预测模型应用于鱼肉中9种PFASs的半定量预测，预测误差倍数为0.79~1.81倍。该模型能够较为准确地进行PFASs的电离效率预测，在无标准品PFASs的可疑和非靶向筛查、风险评估中具有良好的应用前景。

## Abstract (en)

Per- and polyfluoroalkyl substances （PFASs） represent a category of emerging contaminants of global concern in fields such as environmental science and food safety， due to their persistence， bioaccumulative properties and potential toxicity. Although screening methods for PFASs using high resolution mass spectrometry （HRMS） have been developed rapidly， the diversity of PFASs and the absence of standards pose significant challenges for quantitative analysis. In this study， 50 PFASs were analyzed by HPLC-HRMS. The ionization efficiency （IE） was calculated as the slope of the calibration curve. A quantitative structure-activity relationship （QSAR） model was developed employing machine learning to predict the ionization efficiencies of PFASs using PaDEL molecular descriptors. The model enables semi-quantitative estimation of PFASs concentrations in the absence of reference standards by incorporating predicted IE values. Eighteen critical descriptors were selected from a total of 1 444 PaDEL descriptors through the application of recursive feature elimination （RFE）. These selected descriptors encompassed topological descriptors， geometrical descriptors， autocorrelation descriptors， electrostatic and polarity descriptors. These individual descriptors including VE1_Dzv， GATS6i， JGI10， GATS1p and MATS4m were of great importance. Three algorithms including elastic net linear regression， random forest （RF）， and XGBoost were evaluated for model performance. In the elastic net linear regression model， the root mean square error （RMSE） for the training dataset was 0.049 0， and the coefficient of determination （*R*²） was 0.993 0； for the test dataset， the RMSE was 0.163 0， with an *R*² of 0.756 1. In the RF model， the RMSE for the training dataset was 0.163 1， and the *R*² was 0.921 9； for the test dataset， the RMSE was 0.131 6， with an *R*² of 0.840 9. In the XGBoost model， the RMSE for the training dataset was 0.052 1， and the *R*² was 0.992 0； for the test dataset， the RMSE was 0.118 4， with an *R*² of 0.871 3. Nonlinear algorithms of random forest and XGBoost demonstrated superior predictive performance compared to the elastic net linear regression， with XGBoost exhibiting best performance. Random forest， a bagging-based approach， trains individual decision trees independently and aggregates predictions through averaging. In contrast， XGBoost employs gradient boosting methodology， iteratively optimizing the model by sequentially training new trees in order to address residuals from previous iterations. The independent training mechanism of random forest inherently lacks the iterative optimization framework that is characteristic of gradient boosting. Specifically， XGBoost systematically enhances predictive accuracy by generating new trees that target residual errors from preceding models， thereby progressively refining predictive performance. This fundamental difference in optimization strategy enables XGBoost to more effectively correct prediction errors compared to the ability of random forest. Based on the results of a comprehensive evaluation of the three models， the XGBoost algorithm was ultimately selected for its demonstrated performance advantages. The prediction errors of ionization efficiency （IE） for the 50 PFASs were within 1.67-fold， with a median value of 1.04-fold and RMSE of 1.06. The established XGBoost model was further applied for the semi-quantitative concentration prediction of 50 PFASs across concentration gradients， where the prediction errors ranged from 0.12 to 4.90-fold， with a median value of 0.96-fold and RMSE of 0.94. The accuracy of the prediction improved as the concentrations increased. Furthermore， the model was applied to predict concentrations of PFASs in fish tissue. After sample extraction and cleanup using solid-phase extraction， the samples were analyzed using HPLC-HRMS. The concentrations of PFASs were semi-quantified using the predicted IEs， yielding prediction errors ranging from 0.79-fold to 1.81-fold. These findings highlight the robustness of the IE prediction model for PFASs. Notably， the performance of the developed model was better than or comparable to the performance of previous studies. In conclusion， this study introduces a machine learning-based QSAR model for the prediction of ionization efficiency. This approach illustrates the ability to estimate the concentrations of PFASs in the absence of standards， thereby presenting considerable potential for the risk assessment of compounds lacking standards in suspect and non-targeted screening.

全氟及多氟烷基化合物（per- and polyfluoroalkyl substances，PFASs）是一类具有独特理化性质的有机化合物，因其卓越的耐热性、化学稳定性及疏水疏油特性，被广泛应用于工业生产和日常消费品中，如防水防油涂层、电子产品制造、灭火泡沫及食品包装材料等^［1，2］^。然而，这类化合物也具有环境持久性和生物累积性，成为全球关注的持久性有机污染物^［3，4］^。近年来，PFASs在全球环境介质和生物体中广泛检出^［5-7］^，以鱼肉为例，PFASs在鱼肉中被广泛检出^［8］^，且研究发现人体血清中PFASs浓度水平和鱼肉食用量具有关联性^［9］^。PFASs被证实具有潜在的生态毒性和健康危害，包括致癌性、内分泌干扰及免疫系统影响等^［10-12］^。这些问题促使国际社会对其使用和排放实施严格的管控政策，同时推动对替代品和治理技术的研发。

全氟辛烷磺酸盐（perfluorooctanesulfonate，PFOS）、全氟辛酸（perfluorooctanoic acid，PFOA）、全氟己烷磺酸盐（perfluorohexanesulfonate，PFHxS）等传统PFASs的生产和使用受到限制，但仍有大部分结构类似物作为替代品使用^［1］^。目前对于PFASs的分析既包括对特定目标物的靶向分析，也包括可疑和非靶向分析。基于液相色谱-三重四极杆质谱（LC-MS/MS）的靶向分析是目前检测PFASs最主要的分析方法^［13-15］^，LC-MS/MS分析依赖于标准品，由于PFASs标准品种类不足，LC-MS/MS对于无标准品PFASs的分析有一定的局限性。基于液相色谱-高分辨质谱（LC-HRMS）的靶向、可疑和非靶向筛查近年来发展迅速，成为PFASs全面分析的重要工具^［16］^，开发无标准品的PFASs半定量预测方法也是研究的重点。定量构效关系（QSAR）模型是一种基于化学分子结构和性质预测目标化合物行为的数学模型，QSAR模型近年来逐渐应用于高分辨质谱的非靶向筛查半定量预测中，通过化合物的分子质量、电负性、拓扑结构等分子描述符与电离效率等变量之间的数学关系，建立预测模型^［17-19］^。

本研究采用液相色谱-高分辨质谱分离了50种PFASs，获得其电离效率，采用弹性网络、随机森林和XGBoost（Extreme Gradient Boosting）等机器学习算法建立了基于分子描述符和电离效率的QSAR半定量预测模型，比较了不同模型的性能并将所建立QSAR模型应用于鱼肉中PFASs的半定量预测。

## 1 实验部分

### 1.1 仪器与试剂

Vanquish-Q Exactive Plus orbitrap液相色谱-高分辨质谱联用仪（LC-HRMS），美国ThermoFisher公司。Milli-Q超纯水仪，德国Merck公司。ME614S电子天平，德国Sartorius公司；CR21GIII离心机，日本Hitachi公司；KS260型振荡摇床，德国IKA公司；N-EVAP 111氮吹仪，美国Organomation Associates公司。

50种PFASs单标准溶液（50 μg/mL）（表1）及9种PFASs同位素内标，加拿大Wellington Laboratories公司。GBW（E）100740鱼肉粉中9种全氟化合物成分分析标准物质，中国计量科学研究院；甲醇、乙腈，色谱级，美国ThermoFisher公司；醋酸铵，色谱级，德国Sigma公司；OASIS WAX（6 mL， 150 mg）固相萃取柱，美国Waters公司。

**表 1:** 50种PFASs的信息

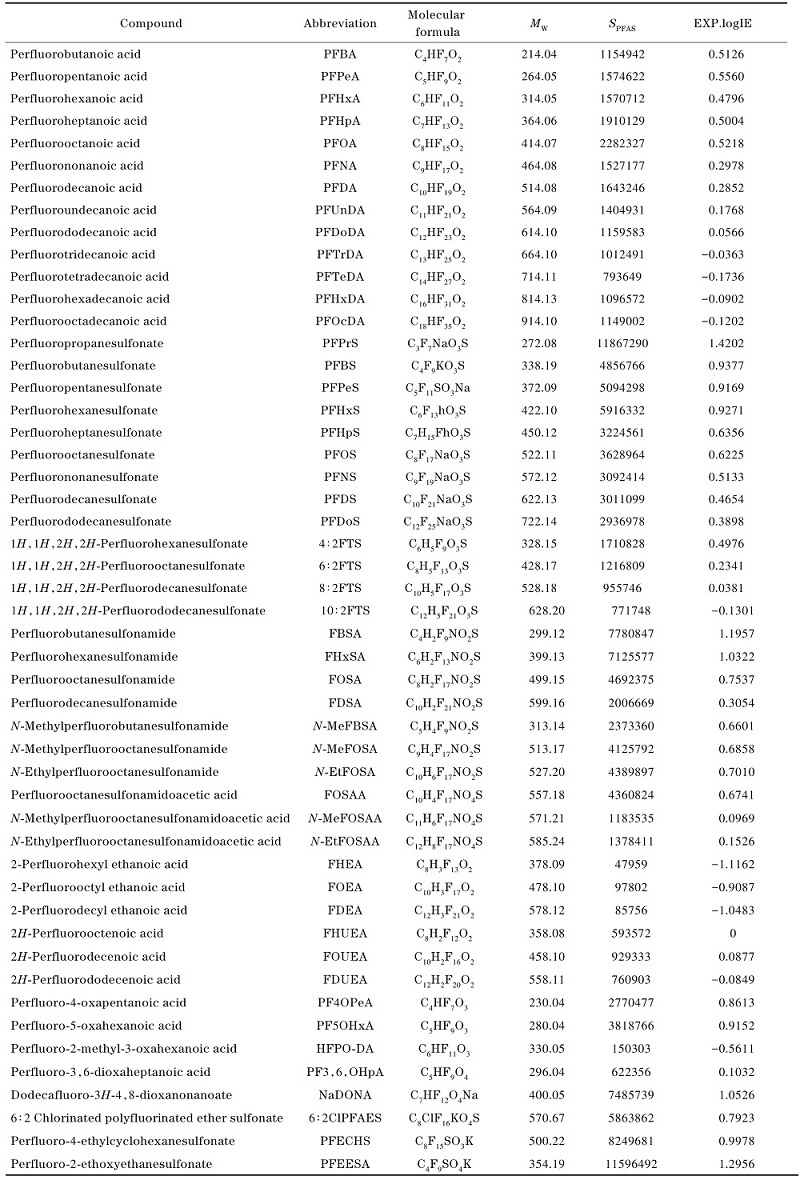

*M*_W_： the molecular weight； *S*_PFAS_： the slope of the calibration curve； EXP.logIE： the relative ionization efficiency determined experimentally.

### 1.2 实验过程

#### 1.2.1 样品前处理

参考实验室前期开发的方法^［20］^，适当优化后，进行鱼肉粉样品的前处理。准确称取0.5 g鱼肉粉样品（精确至0.000 1 g）至离心管，加入1.5 g水复溶至肉浆，立即手摇混匀，涡旋2 min，静置30 min。准确加入同位素内标（精确至0.000 1 g），涡旋2 min，静置30 min平衡。加入10 mL乙腈，振荡提取30 min，超声提取10 min，3 000 r/min离心，转移上清液至离心管。剩余残渣重复提取1次，8 000 r/min离心，合并提取液并氮吹浓缩至3 mL，加入20 mL水稀释。WAX固相萃取柱经4 mL 0.1%氨水甲醇、4 mL甲醇、4 mL水活化。样品稀释液过WAX柱，4 mL 25 mmol/L的醋酸铵溶液（pH=4）淋洗、4 mL水淋洗，抽真空30 min去除水分，固相萃取柱采用4 mL甲醇和4 mL 0.1%氨水甲醇洗脱，收集洗脱液，40 °C水浴下氮气吹至约1 mL，定容至1 mL。溶液在-30 °C冷冻2 h，冷冻离心机10 000 r/min离心10 min，取上清液，过0.2 μm微孔滤膜，转移至进样瓶待测。

#### 1.2.2 分析条件

采用Acquity UPLC BEH C18色谱柱（100 mm×2.1 mm，1.7 μm，美国Waters公司）；流动相由5 mmol/L醋酸铵水溶液（A）和乙腈（B）组成；流速0.2 mL/min；色谱柱温度40 °C；样品温度10 °C；进样体积：5 μL。梯度洗脱程序： 0~3.5 min，90%A~40%A；3.5~9 min，40%A~5%A；9~11 min，5%A；11~11.5 min，5%A~90%A；11.5~15 min，90%A。

采用电喷雾离子源，在负离子模式下进行分析。喷雾电压-2 kV；离子传输管温度350 °C；鞘气流速50 arb；辅助气流速12 arb；辅助气加热温度350 °C；吹扫气流速2 arb；S lens RF 水平80。数据采集采用全扫描-数据依赖采集模式（Full MS-ddMS^2^），一级质谱分辨率为70 000；扫描范围为*m/z* 80~1 000；自动增益控制进入轨道阱中的离子数（AGC target）为1.6×10^5^；最大驻留时间为50 ms；半峰宽设为10 s。ddMS^2^ 扫描参数为分辨率35 000；AGC target为1×10^5^；最大驻留时间为50 ms；TopN设为5；复合碰撞能设置为10、30、70 eV；质量隔离窗口*m/z*为0.4；动态排除时间为10 s。

#### 1.2.3 溶液配制

移取50种PFASs单标准溶液（50 μg/mL），用甲醇配制成100 ng/mL的50种PFASs混合标准溶液。用甲醇对上述混合标准溶液逐级稀释，配制质量浓度为0.2、0.5、1、2、5、10 ng/mL的PFASs系列混合标准溶液，进行液相色谱-高分辨质谱分析。采用一级全扫描的峰面积数据绘制标准曲线，计算标准曲线的斜率作为电离效率（表1）。

#### 1.2.4 QSAR模型构建方法

通过比较各化合物与校正化合物2*H*-全氟-2-辛烯酸（FHUEA）的响应值计算得到其实验相对电离效率（EXP.logIE）。EXP.logIE计算如公式（1）所示，


$$EXP.logIE=log\frac{S_{PFAS}}{S_{FHUEA}}\times \frac{M_{W-FHUEA}}{M_{W-PFAS}}$$ （1）

其中， $S_{FHUEA}$ 、*M*_W-FHUEA_分别为校正化合物FHUEA的斜率和相对分子质量， $S_{PFAS}$ 、*M*_W-PFAS_为各化合物标准曲线的斜率和相对分子质量。本研究PFASs标准曲线斜率基于质量浓度（ng/mL），而在QSAR模型建立中，通常应该以物质的量浓度单位以消除分子质量的影响^［21］^，因此，公式（1）中将质量浓度转化为物质的量浓度。这些通过实验算得的EXP.logIE作为建模时的响应变量，通过PaDEL软件计算得到的各化合物的分子描述符作为输入变量构建QSAR模型。当后续出现没有标准品的全氟化合物即可通过该QSAR模型得到此化合物的相对电离效率（Pred.logIE）。通过预测相对电离效率，可以计算预测的化合物的浓度（ $C_{pred}$ ）， $C_{pred}$ 计算如公式（2）所示，其中 $A_{PFAS}$ 为对应的峰面积。


$$C_{pred}=\frac{A_{PFAS}\times M_{W-FHUEA}}{[10^{Pred.logIE}]\times S_{FHUEA}\times M_{W-PFAS}}$$ （2）

使用PaDEL计算各化合物对应的描述符作为QSAR模型的特征变量，上述计算得到的相对电离效率作为QSAR模型的响应变量。对于特征变量先进行数据预处理和特征清洗，排除当中的异常值和对模型没有贡献的值。再将这50种化合物划分为训练集和测试集，训练集采用不同的算法构建模型，测试集用来评价模型的性能。最终选取模型性能最好的模型用于实际样品的预测。以上QSAR模型的建立通过R和Rstudio软件实现。

## 2 结果与讨论

### 2.1 数据预处理

化合物在ESI-MS中的绝对电离效率受流动相组成、质谱参数、电离源的结构等多种因素的影响^［22-24］^，并且这些参数的影响程度相互关联，其重复性很难控制，因此各化合物的绝对电离效率难以用于浓度预测，因此本研究采用相对电离效率进行浓度预测。本研究选择FHUEA作为校正化合物，一方面，FHUEA质谱响应灵敏度高，在实验的较低浓度水平仍然有很好的质谱信号；另一方面，FHUEA响应具有良好的重复性，其作为校正化合物具有较好的耐受性和稳定性，是良好的电离效率基线指标^［25］^。

PaDEL由Chun Wei Yap发明，用来快速计算化合物从1D到3D的多种分子特征和分子指纹^［26］^。本研究通过PaDEL计算各化合物的2D分子描述符，得到1 444个描述符。因为本研究的PFASs样本只有50个，而计算出的分子描述符数量远大于样本量，引起了“维数灾难”，导致这些数据在空间中分布变得极为稀疏，使得很多算法不再有效，因此，建模之前需要进行特征清洗，首先移除数据集中的缺失值、接近零方差的特征变量和相关系数大于0.8的变量，然后使用递归特征消除（RFE）选取了最佳的特征变量。经过处理后的特征变量有121个，这121个特征变量经过RFE筛选后有18个特征变量保留下来。

### 2.2 电离效率预测模型建立

将50种化合物随机划分为训练集和验证集，训练集中有42种化合物，用于构建模型，验证集中有8种化合物，用于对模型进行外部验证。本研究比较了弹性网络线性回归算法、随机森林及XGBoost非线性回归算法的预测效果。使用训练集决定系数（*R* ^2^）和均方根误差（RMSE）来表征模型的拟合优度，使用测试集*R* ^2^和RMSE表征模型的预测能力。以上功能在R和Rstudio上实现。

弹性网络是一种多元线性回归算法，结合了岭回归和套索回归的优点，适用于处理高度相关的特征或特征数量大于样本数量的情形。预测结果如图1a所示，在训练集上的RMSE为0.049 0，*R* ^2^为0.993 0，测试上的RMSE为0.163 0，*R* ^2^为0.756 1；该方法出现了较严重的过拟合现象，可能是电离效率和特征变量之间并不存在明显的线性关系。

**图1:**
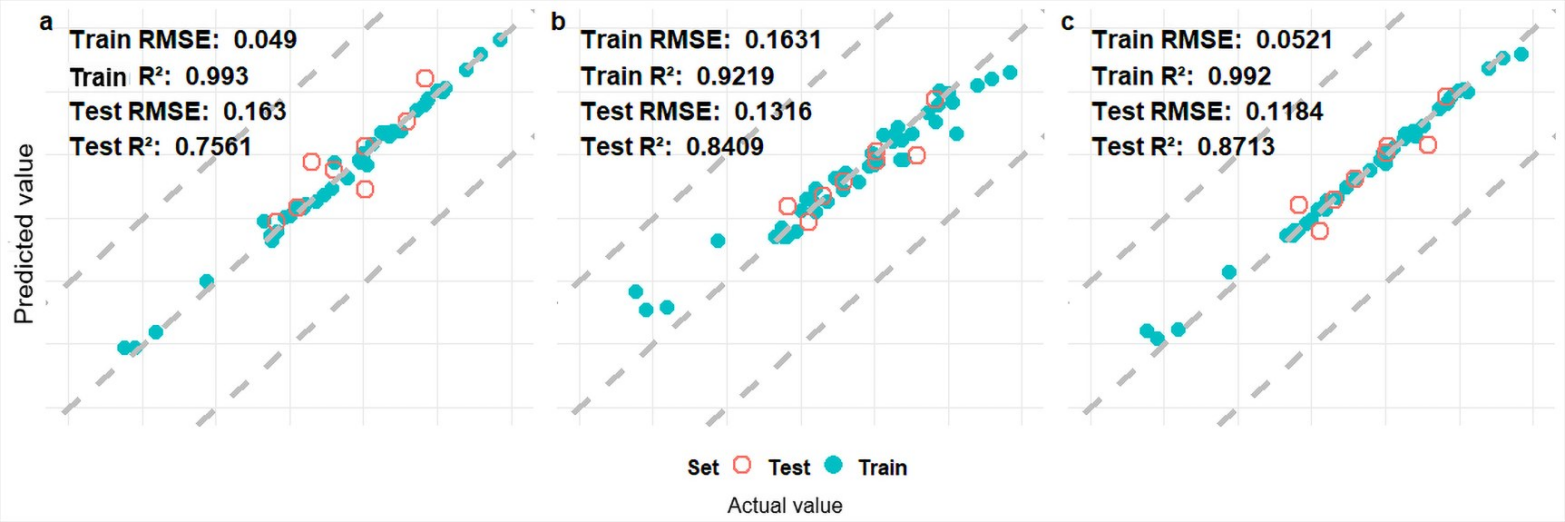
（a）弹性网络、（b）随机森林和（c）XGBoost模型的预测结果

进一步采用随机森林和XGBoost两种多元非线性回归算法进行模型建立。随机森林算法能通过集成多个决策树，每棵树的分裂可以基于不同的特征组合，从而能够有效捕捉变量之间的非线性关系。不像线性回归或其他传统回归方法，随机森林不需要提前假设变量之间的关系，因此特别适用于无法事先定义的复杂数据结构。使用随机森林在训练集上的RMSE为0.163 1，*R* ^2^为0.921 9，测试上的RMSE为0.131 6，*R* ^2^为0.840 9，如图1b所示。XGBoost是一种基于梯度提升树的高效实现，它能通过集成多个决策树的预测来提高精度，同时通过正则化和剪枝避免过拟合。使用XGBoost在训练集上的RMSE为0.052 1，*R* ^2^为0.992 0，测试上的RMSE为0.118 4，*R* ^2^为0.871 3，如图1c所示。

综上，两种非线性方法预测效果均优于弹性网络，且XGBoost的预测效果优于随机森林。随机森林是基于Bagging的方法，每棵树是独立训练的，最终通过平均方式得到预测结果；XGBoost使用梯度提升（Boosting）方法，在每一轮训练中逐步优化模型。由于随机森林模型每棵树都是独立训练的，随机森林的模型学习过程并没有梯度提升那样的迭代优化机制，而XGBoost每次新加入的树是根据前一轮模型的残差来进行训练的，这样能够更精准地纠正之前的预测错误。通过以上3种模型的比较，选择XGBoost算法。

### 2.3 模型机理分析

XGBoost模型最终选取的描述符有18个，主要分为拓扑描述符（topological descriptors）、几何描述符（geometrical descriptors）、自相似性描述符（autocorrelation descriptors）和电性与极性描述符（electrostatic and polarity descriptors）4个类别。拓扑描述符反映分子的拓扑结构特征，描述分子中原子、键和分支的关系。几何描述符为量化分子的几何形状、大小或空间分布。自相似性描述符是基于分子中原子间的物理化学性质的相关性计算。电性与极性描述符描述分子的电性、极性或反应性特征^［27］^。本研究中选择的18个描述符的重要性如图2所示，其中重要性较高的描述符为VE1_Dzv、GATS6i、JGI10、GATS1p和MATS4m。其中VE1_Dzv是基于分子拓扑结构计算的描述符，衡量分子图的电子复杂性和结构特征，它反映了分子中原子和化学键的连接方式和分布。GATS6i是一种基于自相关函数的几何描述符，表示6步长内的分子内部原子间相互作用，且与原子的离子化势加权相关。JGI10是基于分子结构中原子之间路径长度的拓扑描述符，用于量化分子结构的锯齿性或连接的不规则性。GATS1p 是基于分子内1步长的几何自相关性描述符，加权因子为原子的偶极矩。MATS4m是基于4步长的分子内原子间距离的几何自相关描述符，权重为原子的质量^［27］^。这5个描述符都与分子的结构和拓扑相关，并且可以反映分子内原子和键的连接性和空间特征，表明分子整体的结构复杂性和键合模式是影响电离效率的主要因素。

**图2:**
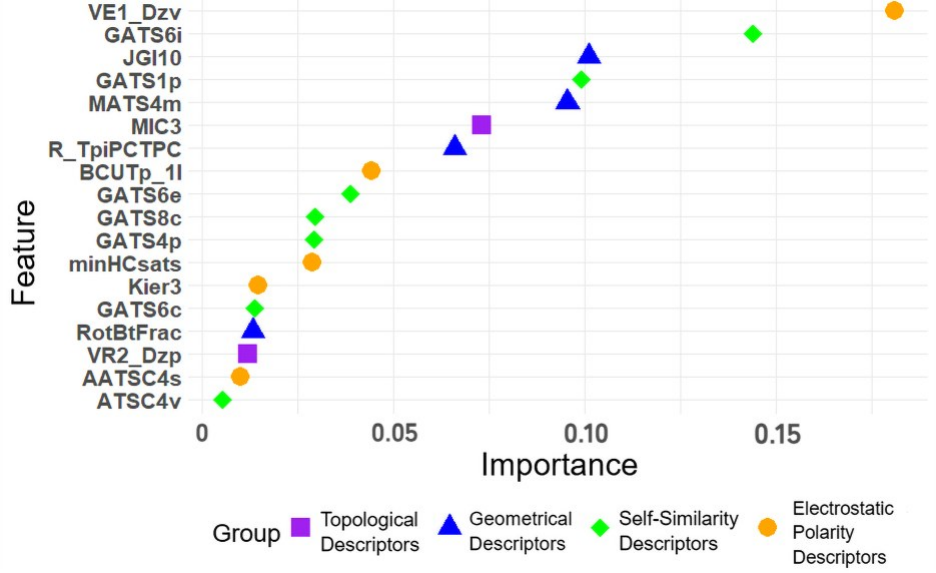
描述符的重要性

### 2.4 预测误差分析

使用公式（3）计算IE的预测误差，其中 $IE_{pred}$ 、 $IE_{actual}$ 分别为通过模型预测的IE和通过公式（1）计算出的实际IE。 $IE_{error}$ 为这两个值的比值，用来表示预测的误差。误差累计图如图3所示，结果表明，模型预测的IE误差都在1.67倍以内，误差中位值为1.04倍，平均值为1.10倍，均方根误差为1.06。误差最大的化合物是2-全氟己烷乙酸（FHEA），发现该化合物IE值仅为0.076 5，是本研究中IE最低的PFASs，其次是6∶2氯代多氟烷基醚磺酸盐（6∶2ClPFAES），误差为1.65倍，该化合物中含有氯原子，碳氯键和碳氟键极性和键能有一定差异，本研究50种化合物仅该化合物含氯原子，模型对该化合物的IE的预测能力相对较差。


$$IE_{error}=\left\{\begin{array}{c} \frac{IE_{pred}}{IE_{actual}}\;IE_{pred}\geq IE_{actual}\\ \frac{IE_{actual}}{IE_{pred}}\;IE_{pred}<IE_{actual} \end{array}\right.$$ （3）

**图3:**
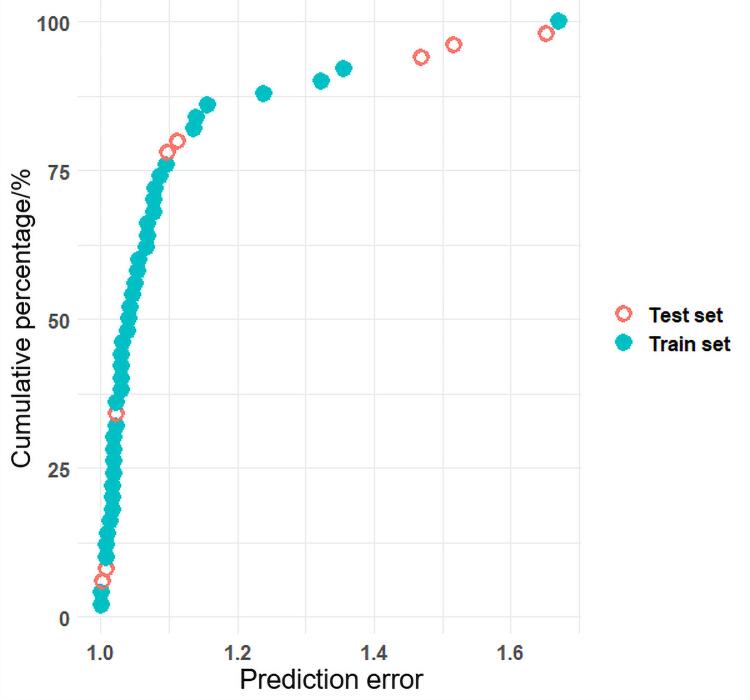
IE预测误差累计图

将预测的电离效率代入公式（2），计算50种不同梯度浓度的PFASs预测浓度（实际质量浓度为0.2、0.5、1、2、5、10 ng/mL）。用公式（4）计算预测误差（error），其中 $C_{actual}$ 为实际浓度。结果表明，总体浓度预测误差倍数在0.12~4.09范围内，误差中位值为0.96，平均值为1.03，均方根误差为0.94。且浓度越高离散程度越低，预测的结果越稳定，如图4所示。图4中的离群点为该梯度浓度下预测结果较差的化合物。


$$Error=\frac{C_{pred}}{C_{actual}}$$ (4)

**图4:**
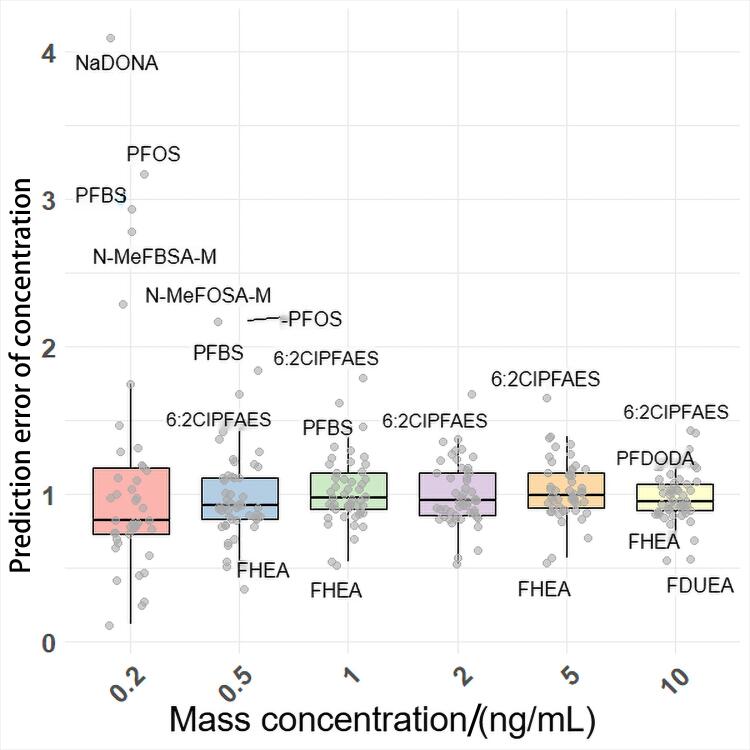
不同梯度浓度的预测误差

对于不同PFASs，各浓度点预测误差中位值为0.53~1.66内，平均值在0.51~1.73，如图5所示。预测误差相对较大的化合物进行分析发现，部分化合物预测浓度整体偏高或偏低，例如，6∶2ClPFAES的预测误差为1.43~1.79，中位值为1.67，平均值为1.62，其预测浓度整体偏高，其误差主要来源于电离效率预测值的误差，FHEA、2*H*-全氟-2-十二烯酸（FDUHA）等也具有相似的误差来源。而对于4.8-二氧杂-3*H*-全氟壬酸盐（NaDONA）、全氟丁基磺酸盐（PFBS）、全氟辛基磺酸盐（PFOS），其IE预测误差为1.14、1.04和1.01，但其0.2 ng/mL和0.5 ng/mL低浓度点的预测误差倍数较大，分析其标准曲线结果发现该两种化合物截距值相对较大，对低浓度预测结果有较大的影响。但本研究整体上对50种PFASs化合物有较好的预测结果。

**图5:**
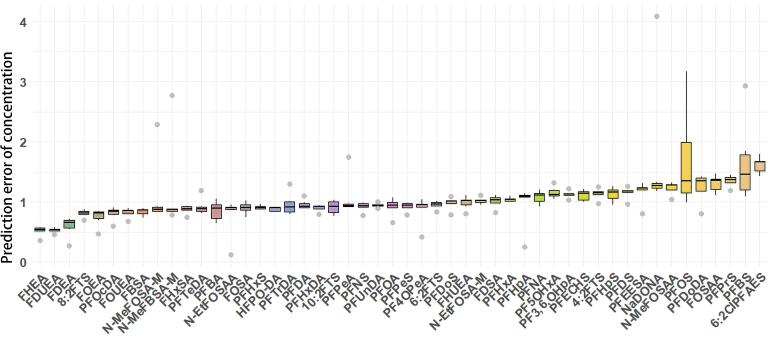
不同化合物的预测浓度误差

### 2.5 模型比较

近年来，QSAR模型逐渐应用于高分辨质谱可疑或非靶向分析中无标准品化合物的半定量预测。本研究采用了XGBoost算法，已有研究中采用的算法主要包括随机森林、梯度提升决策树（xgbTree）、遗传算法-多元线性回归（GA-MLR）、人工神经网络（ANN）等^［28］^。对于PFASs的半定量预测，Hu等^［29］^采用随机森林算法，建立了50种PFASs半定量预测模型，对于电离效率预测误差，Hu等^［29］^模型整体上预测误差最高为2.7倍，中位值为1.1，略高于本研究的预测误差。Lauria等^［18］^在不同类别化合物半定量预测模型的基础上，新加入了33种PFASs，将原模型拓展为能对PFASs进行半定量预测的模型，测试集的最大预测误差为2.3倍，略高于本研究。Liigand等^［17］^构建了药物、代谢物、氨基酸、酚类化合物、杂环化合物等不同类别化合物的电离效率通用预测模型，ESI^+^模式下化合物电离效率的平均预测误差为2.2倍，ESI^-^模式下化合物电离效率的平均预测误差为2.0倍，与该模型相比，本研究也具有良好的预测结果。

### 2.6 模型应用

将本研究建立的模型应用于1种鱼肉标准物质中9种PFASs的半定量预测。经前处理后的样品溶液进行HPLC-HRMS分析后，分别采用同位素稀释质谱法（IDMS）^［20］^和本研究建立的QSAR半定量预测模型进行PFASs定量，同位素稀释质谱法的PFASs定量结果均在标准物质的不确定度范围内（表2）。QSAR模型预测结果与IDMS结果的误差在0.79~1.81倍范围内，证明建立的预测模型具有良好的实际应用价值。

**表 2:** 鱼肉粉中PFASs的预测结果

Compound	Certified value	Uncertainty	IDMS result	QSAR predicted value	Prediction error of concentration
PFBS	3.45	0.45	3.76	4.38	1.16
PFHxS	5.04	0.63	5.17	4.93	0.95
PFOS	6.41	0.93	6.39	6.95	1.09
PFOA	4.47	0.61	4.64	3.65	0.79
PFNA	4.78	0.36	4.59	5.15	1.12
PFDA	4.64	0.49	4.75	4.19	0.88
PFUnDA	4.83	0.69	5.00	4.76	0.95
PFDoDA	4.27	0.55	4.01	7.24	1.81
PFTeDA	3.69	0.51	3.22	3.93	1.22

## 3 结论

本研究开发了一种基于PaDEL描述符和机器学习算法的多类型PFASs半定量预测模型，比较了弹性网络、随机森林、XGBoost算法的预测性能，选取了性能最佳的XGBoost用于电离效率预测。本研究在可疑和非靶向筛查中无标准品化合物的风险评估中具有良好的应用前景，能够为相关研究和应用提供参考。本研究还存在一定的局限性，建模的方法具有良好的适用性，但模型结果只适用于该类型仪器和色谱条件，不同仪器条件应用该方法进行半定量预测时，需进行模型结果的重新输出。未来的研究在建模过程中考虑流动相组成、流动相的比例、pH、黏度等经验描述符能够进一步增强QSAR模型的移植能力和适用性。
